# Déterminants de la pratique « WASH » par les ménages dans la commune de Djougou au Bénin en 2024

**DOI:** 10.11604/pamj.2025.51.70.45787

**Published:** 2025-07-08

**Authors:** Comlan Cyriaque Degbey, Stanislas Isso-issoyirou Bamaï, Judicaël Nounagnon Todedji, Sègla Anthelme Prosper Fassinou, Alphonse Kpozehouen

**Affiliations:** 1 Institut Régional de Santé Publique, Université d'Abomey-Calavi, Ouidah, Bénin,; 2 Clinique Universitaire d'Hygiène Hospitalière, Centre National Hospitalier Universitaire Hubert Koutoukou MAGA, Cotonou, Bénin

**Keywords:** Eau, assainissement, hygiène, déterminants de la santé, Djougou, Water, sanitation, hygiene, health determinants, Djougou

## Abstract

**Introduction:**

plusieurs pays en développement, dont le Bénin, rencontrent des difficultés en matière d'accès à l'eau potable et aux services d'hygiène et d'assainissement (WASH). Djougou, située au nord du Bénin, fait face à des défis similaires. Cette étude avait pour objectif d'analyser les déterminants de la pratique WASH par les ménages dans cette commune en 2024.

**Méthodes:**

il s'agissait d'une étude transversale à volet analytique réalisée entre le 27 mai et le 13 juin 2024. Un échantillon de 165 chefs de ménage a été sélectionné aléatoirement dans deux arrondissements ruraux (Barei et Bariénou) et deux urbains (Djougou I et Djougou II). Les données ont été collectées à l'aide d'un questionnaire et d'une grille d'observation, puis analysées à l'aide du logiciel Epi Info 7.2.5.0.

**Résultats:**

l'étude avait révélé que 49,09% des ménages n'avaient pas accès à des sources d'eau améliorées. Les principales sources d'eau étaient les puits non couverts (49,09%), les forages (27,88%), les puits couverts (12,12%) et l'eau de la Société Nationale des Eaux du Bénin (SONEB) (10,91%). La défécation à l'air libre était pratiquée par 37,58% des ménages. Le niveau d'instruction, le revenu, la distance aux points d'eau améliorés et leur disponibilité influençaient les choix en matière de source d'eau et d'assainissement.

**Conclusion:**

l'accès insuffisant aux services WASH à Djougou est influencé par divers facteurs socio-économiques. Il est crucial d'améliorer l'accès aux infrastructures WASH, en particulier dans les zones rurales, pour répondre aux besoins des ménages et réduire les pratiques d'assainissement inadéquates.

## Abstract (en)

**Introduction:**

several developing countries, including Benin, face significant challenges in accessing safe drinking Water, Sanitation, and Hygiene (WASH) services. Djougou, located in northern Benin, is no exception. This study aimed to analyze the determinants of household WASH practices in this municipality in 2024.

**Methods:**

we conducted a cross-sectional analytical study from May 27 to June 13, 2024. A randomly selected sample of 165 household heads was surveyed across two rural districts (Barei and Bariénou) and two urban districts (Djougou I and Djougou II). Data were collected using a structured questionnaire and an observation grid, then analyzed using Epi Info software version 7.2.5.0.

**Results:**

the study revealed that 49.09% of households did not have access to improved water sources. The main sources of water included uncovered wells (49.09%), boreholes (27.88%), covered wells (12.12%), and water supplied by the National Water Company of Benin (SONEB) (10.91%). Open defecation was practiced by 37.58% of households. Educational level, household income, distance to improved water points, and water availability significantly influenced household choices regarding water sources and sanitation practices.

**Conclusion:**

limited access to WASH services in Djougou is driven by various socio-economic factors. Strengthening WASH infrastructure, particularly in rural areas, is essential to meet household needs and reduce unsafe sanitation practices.

## Introduction

L'eau, l'hygiène et l'assainissement, acronyme anglais *Water, Sanitation and Hygiene* (WASH) constituent une priorité du programme de développement depuis 50 ans, ils comptent parmi les besoins essentiels de l'homme et ont un impact positif sur un certain nombre de ressources essentielles au développement [1]. Ces besoins comprennent l'accès à l'eau potable, l'hygiène, les installations sanitaires, l'agriculture, l'élevage, l'éducation, la protection de l'environnement. Il a été démontré que les interventions WASH améliorent les résultats en matière de santé, en particulier pour les mères, les nouveau-nés et les enfants, à la maison et dans les établissements de santé [2]. Le rapport du programme commun de l'Organisation mondiale de la Santé (OMS) et du Fonds des Nations Unies pour l'enfance (UNICEF) sur le suivi de l'approvisionnement en eau, de l'assainissement et de l'hygiène (JMP) en 2015, montrait que seulement 39% de la population mondiale avait accès à des installations d'assainissement améliorées, soulignant un besoin crucial d'amélioration dans ce domaine [3]. En 2015, à la fin de la période des Objectifs du Millénaire pour le Développement (OMD), 91% de la population mondiale utilisait une source d'eau potable améliorée et 68% utilisaient des installations d'assainissement améliorées [4].

Les États membres des Nations Unies ont aussi adopté en 2015 le programme des Objectifs de Développement Durable (ODD) à l'horizon 2030, dont l'objectif 6 vise à « assurer la disponibilité et la gestion durable de l'eau et de l'assainissement pour tous » [5]. Cinq ans après l'adoption des ODD en 2020, 74% de la population mondiale utilisait des services d'eau potable gérés en toute sécurité et 54% utilisait des services d'assainissement gérés en toute sécurité [5]. Dans les pays en développement, les questions WASH continuent de présenter des défis importants [6]. En effet, un nombre important d'enfants de moins de cinq ans meurent chaque jour de maladies diarrhéiques et d'autres maladies causées par un manque d'assainissement et d'hygiène [7]. Selon l'OMS, cinq (5) maladies d'origine hydrique surviennent souvent lors de conflits dus à la destruction des systèmes WASH [8].

Au Bénin, l'accès à l'eau, à l'hygiène et à l'assainissement n'est pas reluisant. En 2017, l'Enquête Démographique et de Santé (EDS) a montré que 71% des ménages ont accès à une source d'eau améliorée, 87% n'ont pas accès à des installations d'hygiène non améliorées et 80% des personnes disposant d'un endroit pour se laver les mains (55%) n'avaient pas d'eau et de savon [7]. Plusieurs programmes ont été mis en œuvre pour faire face à ces défis. Le programme FDAL a promu des pratiques d'hygiène individuelles et collectives, notamment à travers des initiatives telles que l'Assainissement Total Piloté par la Communauté (ATPC) et la communication pour un changement de comportement [9]. Malgré les progrès, l'accès à des services WASH de base reste très insuffisant en Afrique, en particulier en milieu rural et pour les populations les plus pauvres, avec des impacts sanitaires majeurs [10-12]. Djougou, une ville du Bénin, présente des défis en matière d'approvisionnement en eau potable et de gestion des déchets ménagers, ce qui entraîne des problèmes d'hygiène et d'assainissement [13]. La présente étude se proposait donc d'étudier les déterminants de la pratique WASH dans cette commune en 2024.

## Méthodes

**Figure 1:**
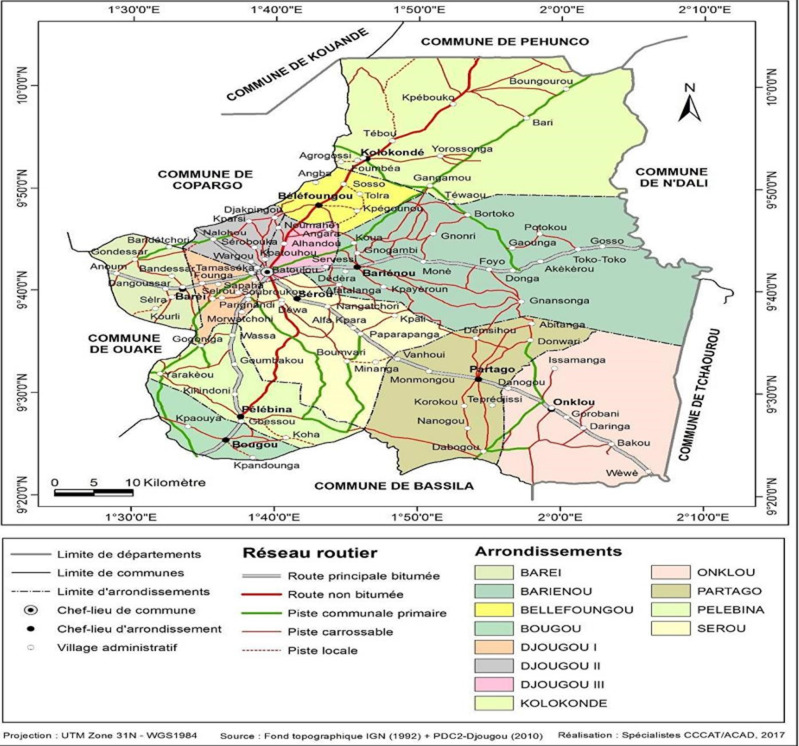
découpage administratif de la commune de Djougou

**Cadre d'étude:** notre étude s'était déroulée dans la commune de Djougou. La commune de Djougou s'étend sur une superficie de 3966 km^2^ et fait partie des quatre communes du département de la Donga au nord du Bénin. Elle est limitée au nord par les communes de Kouandé et de Péhunco, au sud par la commune de Bassila, à l'est par les communes de Sinendé, de N'dali et de Tchaourou, toutes dans le département du Borgou et à l'ouest par les communes de Ouaké, et de Copargo (Figure 1). D'après le Quatrième recensement général de la population et de l'habitation (RGPH4) de 2013, la population de la commune de Djougou est de 267 812 habitants, dont 133 813 hommes (49,97%) et 133 999 femmes (50,03%) [14].

**Type d'étude:** nous avons mené une étude transversale et analytique du 27 mai au 07 juin 2024.

**Population d'étude:** notre étude portait sur les ménages de la commune de Djougou en 2024. Nos cibles primaires étaient les chefs de ménage et celles secondaires étaient les chefs de village ou quartiers de ville.

**Critères d'inclusion:** les répondants lors de notre étude étaient sélectionnés sur la base des critères suivants: être chef de ménage et présent au moment de l'étude; être résident de la commune de Djougou depuis au moins six mois; être âgé de 18 ans et plus et être consentant à participer à l'étude.

**Critères de non inclusion:** n'étaient pas inclus; les individus n'étant pas en mesure de tenir un entretien en raison d'un handicap ou de toute autre raison pouvant expliquer leur incapacité à tenir un entretien.

### Echantillonnage

**Taille de l'échantillon:** la taille de notre échantillon était exprimée en nombre de ménages. Nous avons utilisé la formule de Schwartz pour le calcul de la taille de notre échantillon.

n = (β^2^_a_xpq)/i^2^

n: taille de l'échantillon; ß^2^_a_: valeur qui prend en compte le risque d'erreur accepté (ß^2^_a_ = 1,96^2^); p: prévalence du facteur étudié: 60% de la population de Djougou pratique la défécation à l'air libre [6]; puissance i: précision désirée (i = 8%); q = 1-p. Le calcul effectué nous a donné 144 ménages. Si nous considérons une marge d'erreur de 15% c'est-à-dire le taux de non-répondants, notre taille d'échantillon serait: n = 165 ménages.

**Procédure d'échantillonnage:** nous avons utilisé la méthode d'échantillonnage probabiliste et la technique d'échantillonnage aléatoire simple de degré 3. Le premier degré était le choix des arrondissements et le second a porté sur le choix des quartiers et le troisième degré sur le choix des ménages.

***Premier degré: choix des arrondissements:*** la commune de Djougou est constituée de zone rurale et de zone urbaine. Le choix des arrondissements a été fait par la méthode d'échantillonnage probabiliste et la technique aléatoire simple. Nous avons treize arrondissements et notre étude a été faite dans quatre arrondissements: deux urbains et deux ruraux, soit quatre arrondissements. Nous avons numéroté les arrondissements de 1 à 13 sur des bouts de papier et avons procédé au tirage au sort successif des quatre arrondissements qui feraient l’objet de notre étude. Ont été tirés les arrondissements suivants: Bariénou et Barei en milieu rural, et Djougou 1 et Djougou 3 en milieu urbain. Après avoir sélectionné les arrondissements, nous avons réparti proportionnellement à la taille des ménages de chaque arrondissement, les 165 ménages entre les quatre arrondissements retenus.

***Deuxième degré: choix de villages ou quartiers de ville:*** par la technique d'échantillonnage probabiliste aléatoire simple, nous avons choisi au hasard trois villages ou quartiers de ville dans chaque arrondissement. Les noms de chaque village et quartier de ville ont été inscrits sur un bout de papier et, à l'aide d'un tirage au sort, nous avons tiré les deux villages par arrondissement.

***Troisième degré: choix des ménages:*** pour le choix des ménages, nous avons choisi une direction au hasard en jetant un stylo en l'air à partir du ménage du chef du village ou du quartier de ville. Nous avons suivi la direction indiquée par le stylo lorsqu'il est retombé. Nous avons numéroté sur des bouts de papier les ménages, et à partir d'un tirage au sort, nous avons choisi le premier ménage. Les autres ménages ont été sélectionnés de proche en proche dans cette direction à la suite du premier ménage.

**Techniques et outils de collecte des données:** pour cette étude, nous avons utilisé deux techniques de collecte que sont l'enquête par questionnaire et l'observation avec leurs outils respectifs. Le questionnaire pour les chefs des ménages et la grille d'observation pour les ménages.

**Collecte de données:** les données ont été collectées à l'aide du logiciel Kobocollect.

### Variables

***Caractéristiques socio-démographiques et économiques des ménages:*** âge, sexe, niveau d'instruction, religion, ethnie, profession, niveau de revenu, taille du ménage.

***Pratique des ménages:*** perceptions sur l'eau de boisson, source d'eau de boisson, type de source d'eau, distance jusqu'au point d'eau, temps jusqu'au point d'eau.

***Pratique d'accès à l'hygiène et à l'assainissement par les ménages:*** utilisation des dispositifs de lavage des mains, utilisation des installations sanitaires, type d'installations sanitaires, gestion des déchets solides, gestion des déchets liquides.

### Traitement et analyse des données

***Traitement des données:*** la complétude et la cohérence des données étaient vérifiées. Elles étaient analysées avec le logiciel Epi Info 7.2.6.0. Les données sont organisées sous forme de tableau à l'aide de Microsoft Excel 2016 et le rapport était saisi avec Microsoft Word 2016.

***Analyse des données:*** les mesures statistiques usuelles ont été utilisées en fonction du type de variables: moyennes, écart-type, pourcentages. Les variables quantitatives ont été présentées par leur moyenne suivie de leur écart type. Les variables qualitatives sont présentées sous forme de pourcentages. Pour la recherche des facteurs associés à l'accès aux sources d'eau améliorées et aux services d'hygiène et d'assainissement gérés en toute sécurité, le test de Chi^2^ de Pearson a été utilisé. Il y avait un lien statistiquement significatif lorsque la p-value du test de Khi-2 de Pearson était inférieure à 5%.

**Aspects déontologiques et éthiques:** dans le cadre de notre étude, nous avons reçu une autorisation du ministère de la santé (N°1904-2024/MS/DC/SGM/CNSSP/SA du 27 mai 2024). Les objectifs de l'étude ont été présentés aux chefs de ménage concernés et leur consentement libre et éclairé a été pris en compte. Nous avons garanti la confidentialité à nos enquêtés en collectant les données dans l'anonymat le plus absolu.

## Résultats

**Données socio-démographiques et économiques des ménages:** au terme de notre étude, nous avons eu à enquêter au total 165 ménages. L'âge moyen de nos répondants était de 42,12 ans ± 14,48. Les âges allaient de 18 ans à 80 ans. La majorité des chefs de ménage/représentants des chefs de ménage qui ont participé à notre étude avaient un âge compris entre 35 et 65 ans. Ils représentaient 61,81%. Le sexe féminin et la religion islamique étaient les plus représentés avec un taux de 64,85% et 67,27% respectivement. Concernant le niveau d'instruction, 42,42% de nos enquêtés n'étaient pas instruits. Par ailleurs, l'agriculture et le commerce étaient les activités génératrices de revenus les plus représentées avec 36,97% et 34,55% respectivement. De plus, 66,67% des ménages enquêtés étaient constitués de plus de cinq personnes et plus de 40% des enquêtés avaient un niveau de revenu intermédiaire ou élevé (Tableau 1). La pratique d'accès à l'eau potable par les ménages a relevé que 49,10% des enquêtés utilisent des sources d'eau non améliorées (Tableau 2). Quant à la pratique d'accès à l'hygiène et à l'assainissement par les ménages, 89,09% des enquêtés déversent les déchets liquides dans les rues (Tableau 3).

**Tableau 1:** caractéristiques socio-démographiques des chefs de ménages/représentants

Caractéristiques socio-démographiques	Effectifs (n=165)	%
**Tranche d'âge**		
18-25 ans	16	9,70
25-35 ans	35	21,21
35-65 ans	102	61,82
65 ans et plus	12	7,27
**Sexe**		
Masculin	58	35,15
Féminin	107	64,85
**Religion**		
Christianisme	54	32,73
Islam	111	67,27
**Ethnie**		
Pila-pila	52	31,52
Dendi	55	33,33
Lokpa	45	27,27
Fon	7	4,24
Bariba	6	3,64
**Niveau d'instruction**		
Pas instruit	70	42,42
Primaire	39	23,64
Secondaire	41	24,85
Supérieur	15	9,09
**Profession**		
Agriculteur	61	36,97
Artisan	22	13,33
Commerçant	57	34,55
Fonctionnaire	18	10,91
Sans emploi	7	4,24
**Niveau de revenu**		
Elevé	76	46,06
Faible	21	12,73
Moyen	68	41,21
**Taille du ménage**		
5 ou moins de 5 personnes	55	33,33
Plus de 5 personnes	110	66,67

**Tableau 2:** description de la pratique d'accès à l'eau potable par les ménages

Pratique des ménages	Effectifs (n=165)	%
**Perceptions sur l'eau de boisson**		
Potable	128	77,58
Non Potable	37	22,42
**Source d'eau de boisson**		
Forage	46	27,88
Puits couverts	20	12,12
Puits non couverts	81	49,09
SONEB	18	10,91
**Type de source d'eau**		
Améliorée	84	50,91
Non améliorée	81	49,09
**Distance jusqu'au point d'eau**		
Proche (<100 mètres)	106	64,24
Moyenne (100 à 500 mètres)	36	21,82
Eloigné (>500 mètres)	23	13,94
**Temps jusqu'au point d'eau**		
Moins de 30 minutes	101	61,21
30 minutes à 1 heure	33	20,00
Plus d'1 heure	31	18,79

**Tableau 3:** pratique d'accès à l'hygiène et à l'assainissement par les ménages

	Effectifs	%
**Utilisation des dispositifs de lavage des mains**		
Oui	160	96,97
Non	5	3,03
**Utilisation des installations sanitaires**		
Oui	63	38,18
Non	102	61,82
**Type d'installations sanitaires**		
Latrines améliorées	79	47,88
Latrines non améliorées	24	14,55
Défécation à l'air libre	62	37,58
**Gestion des déchets solides**		
Brûlage	30	18,18
Dépotoirs sauvages	135	81,82
**Gestion des déchets liquides**		
Déversement dans les rues	147	89,09
Caniveaux	4	2,42
Puisard	14	8,48

**Identification des déterminants de l'accès à l'eau potable, l'hygiène et l'assainissement:** pour identifier les déterminants de la pratique WASH par les ménages dans la commune de Djougou, nous avons effectué des tests de Khi deux en croisant les variables indépendantes avec les variables dépendantes: accès aux sources d'eau améliorées, accès aux services d'hygiène et d'assainissement gérés en toute sécurité. Les tests de Khi deux ont montré que les variables suivantes sont significativement associées à l'accès aux sources d'eau améliorées (P-value < 0,05): Le niveau d'instruction, le niveau de revenu, la distance jusqu'aux points d'eau et le manque de sources d'eau améliorées. Par conséquent, ces variables peuvent être considérées comme des déterminants de l'accès aux sources d'eau améliorées par les ménages dans la commune de Djougou (Tableau 4).

**Tableau 4:** déterminants de l'accès ou non aux sources d'eau améliorées

Variables	Khi 2	P-Value
Distance jusqu'au point d'eau	4,3184	0,03
Niveau de revenu	21,6654	0,03.10-4
Manque de sources d'eau améliorées	105,5279	0,00
Niveau d'instruction	8,1552	0,04.10-1

**Accès aux services d'hygiène et d'assainissement gérés en toute sécurité:** les tests de Khi2 ont montré que les variables suivantes sont significativement associées à l'accès aux installations sanitaires gérées en toute sécurité (P-value < 0,05): le niveau d'instruction, le niveau de revenu et le manque d'infrastructures d'assainissement gérées en toute sécurité. Par conséquent, ces variables peuvent être considérées comme des déterminants de l'accès aux installations sanitaires gérées en toute sécurité par les ménages dans la commune de Djougou (Tableau 5).

**Tableau 5:** déterminants de l'accès aux latrines améliorées

Variables	Khi 2	P-value
Niveau d'instruction	23,9341	0,01.10-5
Niveau de revenu	161,0429	0,00
Manque d'infrastructures d'assainissement gérés en toute sécurité	27,1188	0,02.10-5

## Discussion

Dans notre étude, près de la moitié des ménages n'avaient pas accès aux sources d'eau améliorées. Notre étude a confirmé que le niveau d'instruction et le revenu des ménages sont des déterminants clés de l'accès aux sources d'eau améliorées et aux installations sanitaires gérées en toute sécurité. Plus de la moitié des sujets enquêtés étaient de sexe féminin.

**Accès aux sources d'eau améliorées:** au terme de cette étude, l'accès à l'eau potable de base dans les ménages était de 50,91%. Ce résultat est similaire à celui trouvé dans l'étude menée par le Système d'Information pour la Gestion de l'Education au Burkina Faso en 2021 en milieu scolaire. Cette étude avait rapporté que 57,7% des établissements scolaires disposaient d'un point d'eau potable fonctionnel [15]. Notons que notre étude s'était déroulée en communauté et celle menée au Burkina Faso en milieu scolaire. Par ailleurs, selon les statistiques de 2018 du Programme commun OMS/UNICEF sur le WASH, l'accès à l'eau potable de base dans les écoles primaires était de 50,9% [16]. Malgré le fait que ces études aient été faites dans les écoles, les résultats ne diffèrent pas des nôtres. Par ailleurs, 49,09% des ménages dans notre étude n'avaient pas accès aux sources d'eau améliorées. Ces résultats diffèrent de ceux de l'étude du SIGE au Burkina Faso en 2021 qui mettait en évidence que 29% des établissements scolaires ne disposaient pas de points d'eau potable fonctionnels [15]. Cette différence pourrait être expliquée par le fait que cette étude ait été faite dans les établissements scolaires tandis que la nôtre portait sur les ménages.

Les déterminants de l'accès aux sources d'eaux améliorées dans notre étude étaient le niveau d'instruction, le niveau de revenu, la distance jusqu'aux points d'eau améliorés et le manque de points d'eau améliorés. Ces résultats sont similaires à ceux de l'étude menée sur dans le nord-ouest en Ethiopie en 2021 sur les déterminants de la source d'eau, la qualité de l'eau, les perceptions des ménages sur l'hygiène et l'assainissement en milieu urbain où le revenu mensuel, la disponibilité d'installations supplémentaires, l'état de propreté des sources d'eau, la rareté de l'eau et la taille de la famille jouent un rôle crucial dans la détermination des sources d'eau utilisées [16].

**Accès aux services d'hygiène et d'assainissement de base:** la défécation à l'air libre (37,58%) était la pratique d'assainissement la plus répandue dans notre étude. Une étude menée en 2016 au Bénin a montré que 7,30% des ménages dans la commune d'Abomey-Calavi disposaient d'une installation d'assainissement améliorée et que la défécation à l'air libre est pratiquée par 12,7 % des ménages [7]. Ces résultats diffèrent des nôtres du fait que le taux de défécation à l'air libre est presque deux fois plus élevé dans notre étude. Cette différence pourrait être expliquée par le fait que le niveau d'urbanisation est plus évolué à Abomey-Calavi qu'à Djougou. Le niveau d'instruction, le niveau de revenu et le manque de latrines améliorées étaient les déterminants de l'accès aux installations sanitaires gérées en toute sécurité étaient les déterminants de l'accès aux installations d'assainissement de base gérés en toute sécurité. Ces résultats ne diffèrent pas de ceux de l'étude menée en Ethiopie en 2015 où l'âge des participants, leur niveau d'éducation et leur source de revenu sont des facteurs clés [16]. De plus les principaux obstacles identifiés à l'amélioration des pratiques d'hygiène et d'assainissement comprennent la ruralité, le changement climatique, la faiblesse des investissements dans les infrastructures WASH, le manque de connaissances sur les maladies d'origine hydrique et le manque d'engagement communautaire [16]. Ce sont les résultats d'une étude sur l'analyse des impacts de l'urbanisation sur les pratiques WASH en Amérique du Nord en 2019. La différence entre ces résultats et ceux de notre étude pourrait s'expliquer par la représentativité de notre échantillon. De plus, nos résultats diffèrent de ceux d'une étude réalisée par en 2020 dans le premier arrondissement de la commune de Djougou où le mode traditionnel de gestion et les difficultés d'hygiène et d'assainissement des infrastructures hydrauliques l'absence d'implication des autorités locales dans la gestion et la perte de temps au niveau des sources d'eau étaient les déterminants de l'accès à l'eau potable. Cette différence serait due aux changements contextuels survenus entre 2022 et 2024. Nos résultats diffèrent aussi de ceux de l'étude menée par Tseole *et al*. en 2022 [17] en Afrique du Sud où la ruralité, les changements climatiques, la faiblesse des investissements du secteur WASH, le manque de connaissances sur les maladies hydriques et le manque d'engagement communautaire étaient les déterminants de la pratique WASH. Ces différences s'expliquent par les changements contextuels.

S'agissant des limites et des potentiels biais, nous pouvons dire que cette étude a utilisé une conception transversale ce qui ne permet de tirer aucune conclusion sur la relation de cause à effet des pratiques des ménages et leurs déterminants. La méthode d'échantillonnage a été probabiliste et la technique était le sondage aléatoire à 3 degrés. La collecte des données été effectuée par des enquêteurs formés afin de minimiser les biais de sélection. Un biais d'information aurait pu être introduit car les données ont été obtenues par déclaration des enquêtés mais les enquêteurs ont pris le temps de bien expliquer le bien-fondé de l'étude aux enquêtés afin de réduire si possible ce biais. Malgré ces limites ou insuffisances, celles-ci n'entachent pas la crédibilité et l'originalité de cette étude.

## Conclusion

Les travaux menés auprès de 165 ménages dans quatre arrondissements de la commune de Djougou en 2024 nous ont permis d'étudier les déterminants de la pratique WASH par les ménages dans cette commune en 2024. Cette étude a révélé que le niveau d'instruction, le niveau de revenu, la distance jusqu'aux points d'eau améliorés et le manque de points d'eau améliorés sont les déterminants de l'accès aux sources d'eau améliorées d'une part et que le niveau d'instruction, le niveau de revenu et le manque de latrines améliorées sont les déterminants de l'accès aux installations sanitaires gérées en toute sécurité. Des bonnes pratiques WASH contribueraient à réduire la morbidité et la mortalité des maladies hydriques et celles liées au défaut d'hygiène et d'assainissement. De ce fait, il est d'importance capitale de mener une étude sur les facteurs qui entravent l'accès aux services d'eau améliorés et aux services d'hygiène et d'assainissement gérés en toute sécurité par les ménages dans la commune de Djougou.

### Etat des connaissances sur le sujet

- Des nombreuses études en Afrique de l'ouest, comme celle du SIGE en 2021, ont montré que l'accès à des sources d'eau potable et à des infrastructures d'assainissement reste limité, surtout dans les zones rurales;
- Des études, notamment en Éthiopie [7], ont identifié des facteurs comme le niveau de revenu, la distance aux sources d'eau, et la disponibilité des installations comme déterminants principaux de l'accès aux services WASH;
- Des facteurs comme la ruralité, les conditions économiques et climatiques, ainsi que le manque d'engagement communautaire sont souvent des obstacles au bon fonctionnement des services WASH.

### Contribution de notre étude à la connaissance

- Notre étude a révélé que les femmes sont plus représentées dans les pratiques WASH à Djougou (61%), car elles sont généralement responsables de la gestion de l'eau dans les ménages; c'est un point souvent évoqué mais peu quantifié dans les études antérieures;
- Nous avons trouvé que près de la moitié des ménages (49,09%) à Djougou n'ont pas accès à des sources d'eau améliorées; c'est un résultat important, surtout comparé aux études sur les écoles qui montrent un accès plus important;
- En plus des déterminants traditionnels comme le revenu et l'éducation, nous avons montré que la disponibilité de points d'eau améliorés et de latrines est un facteur clé à Djougou, particulièrement en milieu rural.

## Remerciements

Les auteurs remercient les acteurs de la santé, de la municipalité et ceux de la communauté de la commune de Djougou pour leur contribution à la réalisation de cette étude.
